# Micro-computed tomography of termite gut anatomy: effects of staining regime on resolvability

**DOI:** 10.7717/peerj.21334

**Published:** 2026-07-03

**Authors:** Sean J. Blamires, Travers Sansom, Sebastian Oberst

**Affiliations:** 1 Centre for Audio, Acoustics and Vibration, School of Mechanical and Mechatronic Engineering, University of Technology Sydney, Sydney, New South Wales, Australia; 2 School of Biological, Earth and Environmental Science, University of New South Wales, Sydney, New South Wales, Australia

**Keywords:** Micro-computed tomography, Tissue staining, Termite anatomy, *Nasutitermes exitiosus*, Image contrasting, Tissue contrast, Insect microanatomy, Internal organ visualization

## Abstract

Examining anatomical features of exceptionally small animals presents significant challenges for researchers. Micro-Computed Tomographic (µ-CT) scanning coupled with target tissue staining has enabled many previously unseen structures of small insects to be mapped and measured in detail. Here we investigated the effectiveness of three staining regimes: Lugol’s iodine solution (LS), LS plus 1% phosphotungstic acid (PTA), and LS plus 2% PTA, in enhancing the resolvability of µ-CT scans of the gut organs of the termite *Nasutitermes exitiosus*, which has protist-independent digestion. We compared pixel intensity and probabilistic tissue contrast (PTC) across the three staining regimes to determine their ability to enhance image contrasts and improve the resolvability of the internal structures. We measured and compared volume and surface area of the termite foregut, midgut, hindgut, salivary glands and cephalic ganglion (brain). Our results showed LS plus PTA significantly improved image resolution for these termite organs, with LS combined with 2% PTA being the most effective staining regime for enhancing pixel intensity and PTC of most of the organs studied. LS plus 1% PTA resulted in an improved image quality but was generally less effective than LS plus 2% PTA. The LS alone regime gave the lowest resolvability, especially for less radio-dense structures like the salivary glands and midgut. Our results suggest that staining with LS plus 2% PTA generally offers significant image enhancement of µ-CT derived images of microscopic insect organs. This approach can be applied more broadly to significantly improve visualizations of the internal anatomy in other small arthropods and soft-bodied invertebrates, facilitating future research in comparative morphology, developmental biology, and functional anatomy.

## Introduction

Anatomical traits of ecosystem engineering animals such as the gut of termites, crabs, and earthworms have important roles in nutrient and energy recycling at microhabitat through to landscape scales, yet they remain largely underexplored (Harvey & Gutberlet, 1995; Erwin, 2008; Jouquet et al., 2011). For termites specifically, a specialised digestive physiology and gut morphology have evolved in conjunction with a symbiotic microbiota that facilitates the breakdown of cellulose and other recalcitrant organic materials found in wood, leaf litter, and soil (Fujita et al., 2010; Bignell, 2011; Peterson & Scharf, 2016). These adaptations help termites to recycle carbon and nitrogen with the effect of improving soil quality within their subterranean ecosystems (Hopkins et al., 1998; Bagyaraj, Nethravathi & Nitin, 2016). An exploration of the gut morphology of termites, accordingly, provides insights into how environmental pressures can shape internal anatomical features and, in turn, how these features allow ecosystem engineers to adapt to, and influence, their surroundings (Eggleton, 2011).

The gut of termites has three distinct parts: the foregut, midgut and hindgut (Eggleton, 2011; Scharf, 2020). Ingested food initially passes through the foregut wherein the crop and gizzard grind up the cellulose-rich material into particles of less than 50 μm. The midgut is where nutrient absorption is initiated (Terra & Ferreira, 2020). The hindgut then acts somewhat like a bioreactor, as it is where enzyme-producing bacteria and protozoans (in the case of “lower” termites) are found (Scharf, 2020). The hindgut also contains the rectum and is of variable length depending on the digestive capacities of the foregut and midgut (Eggleton, 2011). Nitrogenous wastes are removed by the malphigian tubules which lie at the junction between the midgut and hindgut (Scharf, 2020; Terra & Ferreira, 2020). Structural adaptations of the gut epithelium, such as the development of columnar cells and basal membrane invaginations, enhance nutrient absorption and ionic transport across the gut (Arias, Chevalier & Roisin, 2020; Scharf, 2020; Terra & Ferreira, 2020).

Termites that initiate cellulose digestion in the foregut and midgut express endogenous β-glucanases (Watanabe, Noda & Tokuda, 1998; Tokuda et al., 1999, 2012). However, complete digestion of cellulose still depends on the intricate interplay between host enzymes, gut microbiota, and digestive physiology, which together enable the efficient extraction of micro- and macronutrients from cellulose-rich materials (Bignell, 2011; Brune & Ohkuma, 2011; Scharf, 2020; Arora et al., 2022).

The evolutionary transition from a reliance on both bacterial and protist gut symbionts to solely bacterial gut symbionts is considered the most significant step in termite evolution (Bignell, 2000; Tai et al., 2015; Waidele et al., 2017; Carrijo et al., 2023). As wood feeders, the hindgut of protist-dependent termites contain unique microbial communities that are capable of efficient cellulose degradation and nitrogen recycling (Brune & Ohkuma, 2011; Brune, 2014; Dar et al., 2022). Other adaptations include segmentation across the midgut and hindgut (Nalepa, 1994). These features extenuate the interplay between gut morphology, its microbial symbionts, and the termite’s ecological functionality (Bignell, 2011; Arora et al., 2022).

Protist-independent termites (family Termitidae) lack the flagellated protists found in the so-called protist-dependent termites (*i.e*., the non-termitid families) and have, in contrast, a wider variety of bacterial symbionts in their hindgut to assist with digestion. Among these, species of the genus *Nasutitermes* serve as important models for understanding bacterial-mediated cellulose degradation. Wood-feeding *Nasutitermes* species do not contain cellulose-fermenting protozoa within their hindgut (Warnecke et al., 2007) but possess a highly diverse and compartmentalized gut microbiota comprising of more than 1,000 bacterial phylotypes organized into distinct communities across the digestive tract (Tokuda et al., 2018). Of particular importance are the wood digesting bacteria colonizing the dilated hindgut paunch (Tokuda et al., 2018).

Despite its evident importance, detailed morphological studies of the digestive tracts of small insects, such as *Nasutitermes exitiosus* (whose average body length ~<5 mm), using conventional microscopic techniques have been limited due to difficulties in the capture and visualization of features (Yara, Jahana & Hayashi, 1989; Tokuda, Yamaoka & Noda, 2000; Tokuda et al., 2001). Moreover, dissecting and immunostaining the internal organs using exceptionally small insects is destructive to samples (Fujita et al., 2010). High-resolution imaging techniques on the other hand enable for non-invasive, three-dimensional visualizations of exceptionally fine-scaled anatomical features (Sogawa et al., 2020; Craig et al., 2022; Kallal & Wood, 2022; Ma et al., 2022; Savvidis et al., 2022).

Procedures, such as scanning electron microscopy (SEM), X-ray photoelectron spectroscopy (XPS) and phase contrast computed tomography (PC-CT) imaging, require either the application of a destructive coating, a synchrotron-generated radiation source, or vacuum-sealed conditions for execution, and even then they provide rather superficial surface-level visualizations (Hanus et al., 2007; Killiny & Brodersen, 2022; Mensa et al., 2022; Savvidis et al., 2022). Alternatively, micro-computed tomographic (μ-CT) imaging creates quality three-dimensional images without destructive preparation or a synchrotron radiation source, and is the preferred non-invasive method for the detailed examination of the internal tissue structures of exceptionally small insects (Smith et al., 2016; Gorb et al., 2019; Kallal & Wood, 2022; Mensa et al., 2022; Sansom et al., 2022). As such, high-resolution µ-CT scanning, coupled with advanced image rendering computational methods, has enabled the detailed mapping of the tracheal system, mandibular muscles, nerves, and alimentary canals, of a range of insects (Greenlee et al., 2009; Greco et al., 2014; Iwan, Kamiński & Raś, 2015; Ha et al., 2017; Raś, Iwan & Kamiński, 2018; Swart et al., 2016; Alba-Alejandre, Alba-Tercedor & Vega, 2019).

Radiodensity refers to the degree to which tissues can absorb X-rays during imaging. In the context of visualizing insect organs using μ-CT, radiodensity plays a crucial role in determining how clearly internal structures can be distinguished (Christiansen, 2016; Smith et al., 2016; Sansom et al., 2022). Some insect tissues, including the digestive organs of termites, have exceptionally low radiodensities, which severely limits their resolution and hampers detailed morphological examinations using μ-CT imaging (Bartling et al., 2007; Hanus et al., 2007; Karch et al., 2016).

The use of contrast-enhancing staining agents such as Lugol’s iodine (LS) and phosphotungstic acid (PTA) may become necessary when imaging low radiodensity tissue, which is difficult to resolve using conventional μ-CT imaging (Gignac et al., 2016; Krings et al., 2017). A recent study (Sansom et al., 2022) found that a staining regime of LS followed by 2% PTA facilitated the attainment of µ-CT imagery clear enough to visualise and measure the low radiodensity tissue of the haemolymph chamber and subgenual organ (SGO) in the legs of the termite *Nasutitermes exitiosus*. We accordingly aimed herein to determine whether applying the contrasting agents LS and PTA under carefully optimized conditions renders it possible to better resolve low radiodensity, hence indistinct, termite gut, and associated, features including the boundaries of the foregut, midgut, hindgut, salivary glands, and brain. We hypothesised that the addition of PTA to LS would enhance image contrast and organ delineation, although we expect that the degree of improvement would vary among tissues depending on their intrinsic composition, radiodensity, and affinity for the different staining agents. We anticipated that more radio-dense tissues, such as the cephalic ganglion and hindgut, would respond more effectively to PTA enhancement, whereas less radio-dense or fluid-filled organs such as the midgut and salivary glands might show limited contrast gains at higher PTA concentrations.

## Materials and Methods

**Figure 1:**
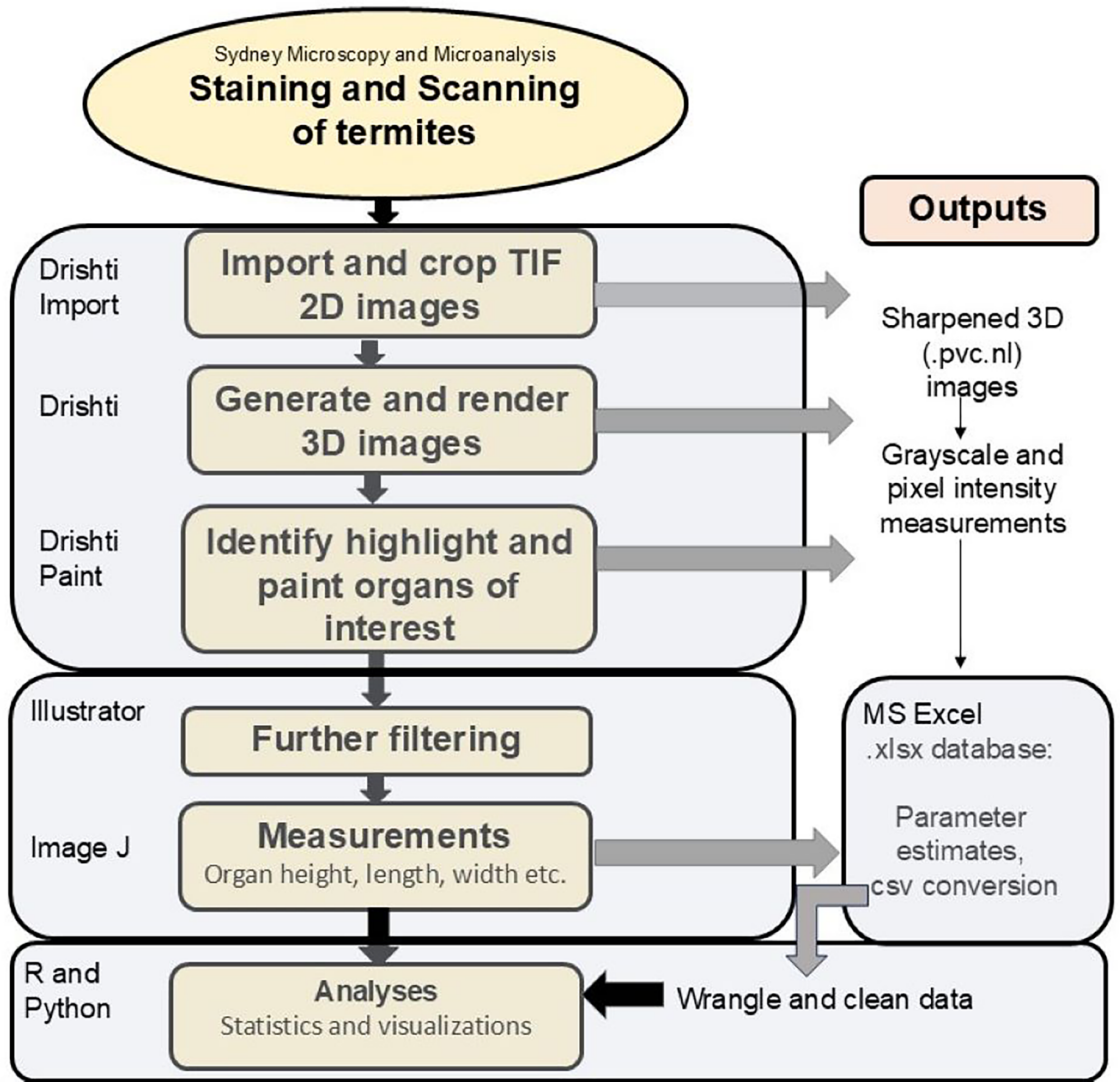
The full methodological pipeline, from scans to processing to outputs, followed herein. Individual components of the pipeline are outlined in detail in the ‘Methods’ section. Grey boxes wrapped around information is to show that each of the processes occurred within individual computing environments and are identified within boxes.

The full methodological pipeline, from staining and scanning of the termites to data analyses and their interpretations, is shown in Fig. 1.

### Staining and scanning of insects

Micro-computed tomographic scans of *N. exitiosis* soldiers of varying resolution, under one of three staining regimes: (1) LS alone, (2) LS plus 1% PTA, and (3) LS plus 2% PTA, were performed at the Sydney Microscopy and Microanalysis Centre, at the University of Sydney, Australia. These stains are identical to those used by Sansom et al. (2022), the difference here is that they are specifically being applied to visualize organs of the digestive tract.

Specimens used for these scans were collected, prepared, and stained, as outlined by Sansom et al. (2022). Scan settings and spatial resolutions were set according to the sample’s distance from the X-ray focal point (~0.45 mm) (Alba-Alejandre, Alba-Tercedor & Vega, 2019; Sansom et al., 2022). The resulting images were stored on the University of Technology Sydney (UTS), Faculty of Engineering and Information Technology’s (FEIT) data server. Those images with the highest resolution (voxel size ~0.45 µm) were used herein to test the effect of staining regime on the resolvability of the hindgut, midgut, foregut, salivary glands, and cephalic ganglion of *N. exitiosus*.

### Image processing and measurements

We superimposed coordinates generated at selected locations across each of the images to facilitate the calculation of volume and cross-sectional area of the morphological features as per Sansom et al. (2022) and Stelzera & Krenkel (2022).

First, three image sets, each containing approximately 1,000 images, depending on the set, of two-dimensional 16-bit TIF images were obtained from the UTS data server. Each of these image sets was an output of a µ-CT scanned termite that had been treated with one of the three staining regimes. The image sets were uploaded into the Import package (Drishtiimport) of the program Drishti (Limaye, 2012), wherein they were cropped and the grey scale contrast (ascertained by the calculated voxel density value) maximized by selecting the highest contrasting components. This process removed artefacts and any background noise that did not contribute to the visual quality of the subsequent outputs.

From each of the two-dimensional images, three-dimensional outputs were generated within Drishti (Limaye, 2012). Upon reading the files and pre-setting the voxel sizes of the output images, a single dense 3D block became visible. This represented the visual output for an insect and the surrounding embedding matrix. The component of the image comprising the embedding matrix was thereupon identified and minimized using Drishti’s voxel resolving tools (Limaye, 2012).

Further refinements and sharpening of the images were attained by isolating images across the *X*, *Y*, and *Z* directions and adjusting the centre point, lighting, shadow contrast, colouration, and transparency settings to reduce excess shading. The bounding box limits were adjusted to digitally ‘dissect’ the images and high-resolution photographs of the internal organs thereupon taken and pixel intensity and associated grayscale values of the cephalic ganglion were measured.

### Organ identification and contrast measurement

**Figure 2:**
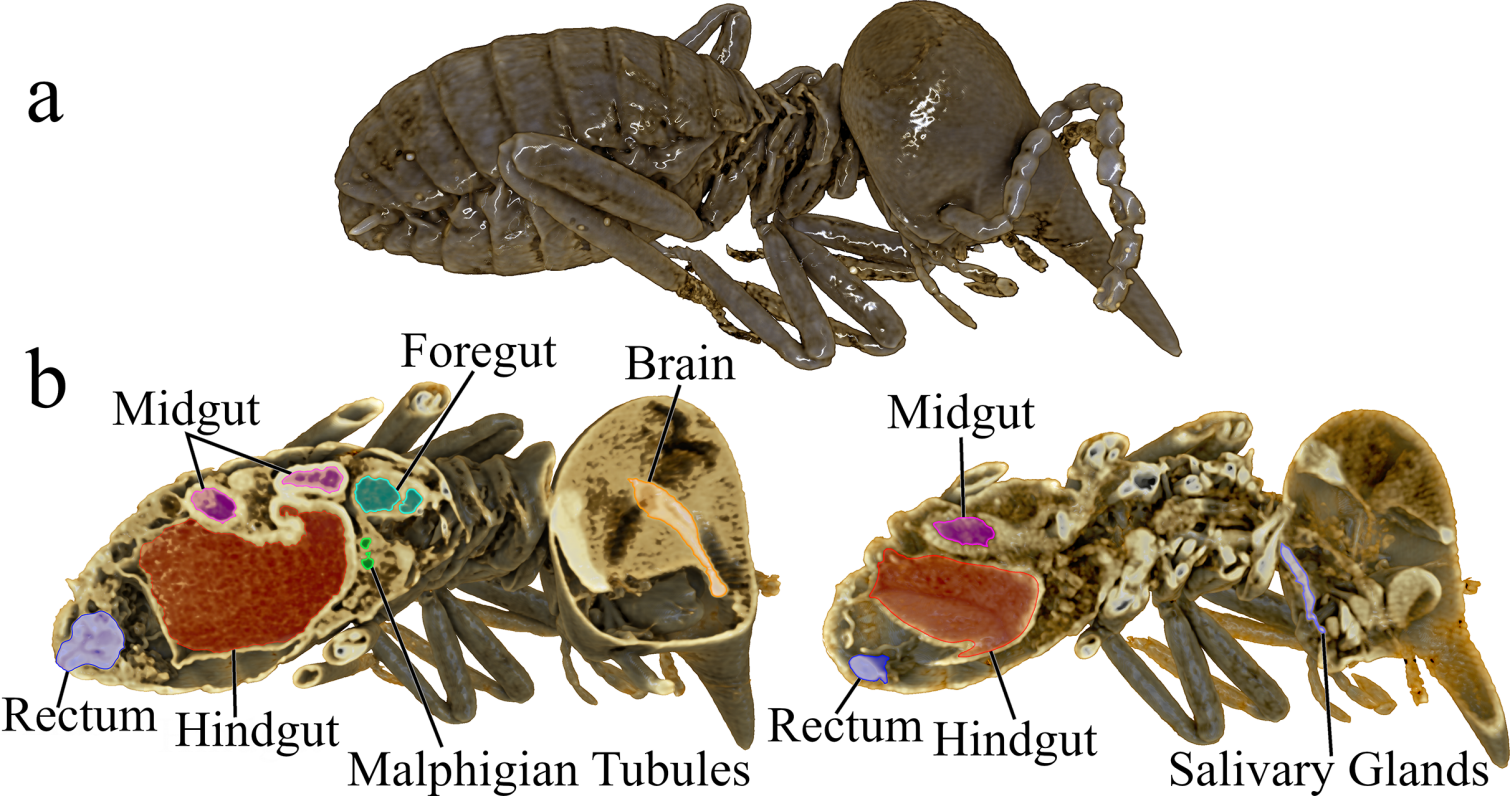
(A) Raw images derived from a high-resolution scan that was stained using Lugol’s Iodine Solution (LS) alone. (B) Location of the morphological features: hindgut, midgut, foregut, rectum, Malpighian tubules, and brain, as were identified from those scans.

The salivary hindgut, midgut, foregut, salivary glands, and dorsal brain of *N. exitiosus* were initially identified across images as depicted in Fig. 2.

The Malpighian tubules of *N. exitiosus* appeared as a clustered mass adjacent to the midgut–mixed segment/hindgut boundary (Hellemans et al., 2024). The structure we annotated as the cephalic ganglion (brain) occupies a region overlapping the expected position of the frontal gland reservoir. The thoracic salivary glands were detected, as per Costa-Leonardo, Bueno da Silva & Teixeira Laranjo (2023), as paired lobed masses in the thorax with an acinar architecture.

**Figure 3:**
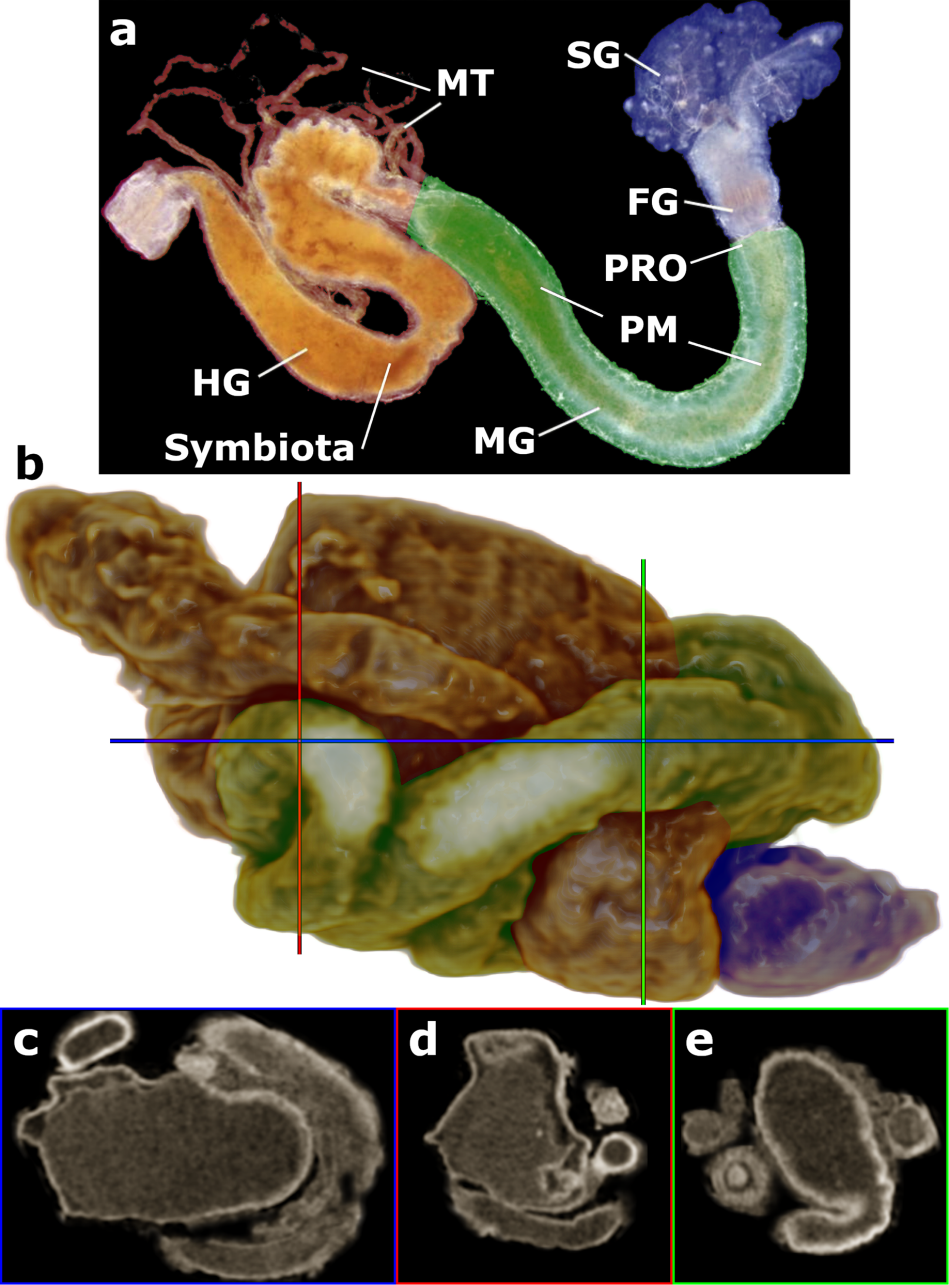
(A) Segmented gut as seen under a light microscope, where the salivary glands (SG) and foregut (FG) is coloured x, the midgut (MG) coloured y, and the hindgut (HG) coloured z. (B) Segmented gut as seen by μ-CT imaging using the same colour scheme to represent the three gut segments as described for (A). (C) Two dimensional image of the termite gut taken from a longitudinal slice (along the purple plane in image (B), hence why there is a purple border around the image). (D) Transverse slice taken near toward the rectum (red plain in image (B)), thus mostly through the hindgut, and (E) transverse slice taken toward the head, through mostly foregut (green plain in image (B)). Abbreviations: SG, salivary gland; FG, foregut; PRO, proventriculus; PM, peritrophic matrix/membrane; MG, midgut; MT, Malpighian tubules; HG, hindgut, as identified according to Scharf (2020).

Unlike in light microscopy where organ topology is manipulated through dissection, spreading and staining the tissue across an imaging surface (Fujita et al., 2010; Costa-Leonardo, Bueno da Silva & Teixeira Laranjo, 2023; Hellemans et al., 2024), all the termite alimentary tract’s natural folds are fully preserved when μ-CT scanning. As the tract is folded and subdivided into proctodeal segments, the forgut, midgut, and/or hindgut may at times resemble each other or even other organs within the examined images (Costa-Leonardo, Bueno da Silva & Teixeira Laranjo, 2023; Hellemans et al., 2024). Identifying the gut compartments is challenging using μ-CT imaging because the abdomen and gut of termites are of extremely low radiodensity. Given such limitations, we delineated the three gut regions from the surrounding tissues by examining the entire μ-CT image set and carefully manually painting out all non-gut background. This isolated the individual gut segments before their full three-dimensional reconstruction. An image stack was then generated and imported into DrishtiImport to verify that the entire alimentary tract had been consistently captured. Following verification, the images were processed using MATLAB to smooth out the layer masks and minimise minor inter-slice jitter as a result of the manual isolation of the gut from other components. The final image stack was imported back to DrishtiImport and then into a Drishti environment, whereupon further three-dimensional rendering, imaging, and viewing of the gut segments were done (see Fig. 3). From these images, pixel intensity and the associated grayscale values of the hindgut, midgut, foregut, and salivary glands, were measured. At least twenty measurements were made of each of the organs across the most resolvable one-hundred or so images from each set. Therefore, we made approximately 2,000 measurements per staining regime.

Probabilistic tissue contrast (PTC) is a measure of the probability that a given organ can be positively identified. It provides a statistically grounded method for assessing how effectively staining protocols enhance tissue differentiation. This is particularly crucial for small and densely packed organs (Swart et al., 2016). We, accordingly, measured grayscale values across and between each of the organs to estimate PTC using calculations outlined by Swart et al. (2016). Repeatedly measuring PTC for each organ in each image while varying the organ scan lengths (Sansom et al., 2022) allowed us to ascertain the influence of organ scan length, in pixels, on the ability of each stain to attain an adequately contrasting image for each organ of interest.

Once visually derivable images were attained, high resolution examinations of the external and internal features were made and all selected images saved as image files for further filtering within Adobe Illustrator before exportation to Image J for measurement (Rueden et al., 2021). We measured the length, width and height (*n* = 20 dimensions per organ per image set, 30,000 measurements in total) of the hindgut, midgut, foregut, salivary glands, and brain. From these, each organ’s volume and cross-sectional area measured (Shaha et al., 2013; Sansom et al., 2022). These processes were repeated three times per image set.

### Data handling and statistical analyses

A dataset of the 30,000 measurements was compiled and saved as a comma delimited file for exportation to the appropriate R (*e.g*., dbplyr, DataExplorer, gpa,) and Python (Numpy, Pandas, Scipy) packages for data processing and analyses (Fig. 1).

Distributional, quartile-quartile, and correlation analyses were performed across staining treatments, and between images within treatments, to determine data distributions and to check for autocorrelations between variables. The relative importance of each of the measured variables were assessed across tangent space using Principal Component Analyses (PCA) (Botton-Divet et al., 2016).

As the data for all variables measured were not normally distributed across treatments and there were autocorrelations among and within multiple variables (see Figs. S1–S3), we conducted Kruskall-Wallis and Dunn’s *post-hoc* tests to compare the influence of the staining method on PTC for each of the organs examined (Kassambara, 2017). Tissue volumes and cross-sectional areas were compared across treatments using multivariate analyses of variance (MANOVA) upon log transformation of the data, or by using equivalent non-parametric (*e.g*., Kruskall-Wallis) analyses when log transformation failed to normalize the data (Iwan, Kamiński & Raś, 2015; Krings et al., 2017).

## Results

Examinations of the distribution, quartile-quartile, and correlation analyses (Figs. S1–S3) suggest that the signal-to-noise-ratio was enhanced, thus each of the organs became more clearly resolvable, when either 1% or 2% PTA was included with Lugol’s tissue staining LS. Our corresponding statistical analyses nevertheless showed there to be, depending on the parameters considered, variation in how well the different stains enhanced the resolvability of each of the different organs.

### Principal component analyses

**Figure 4:**
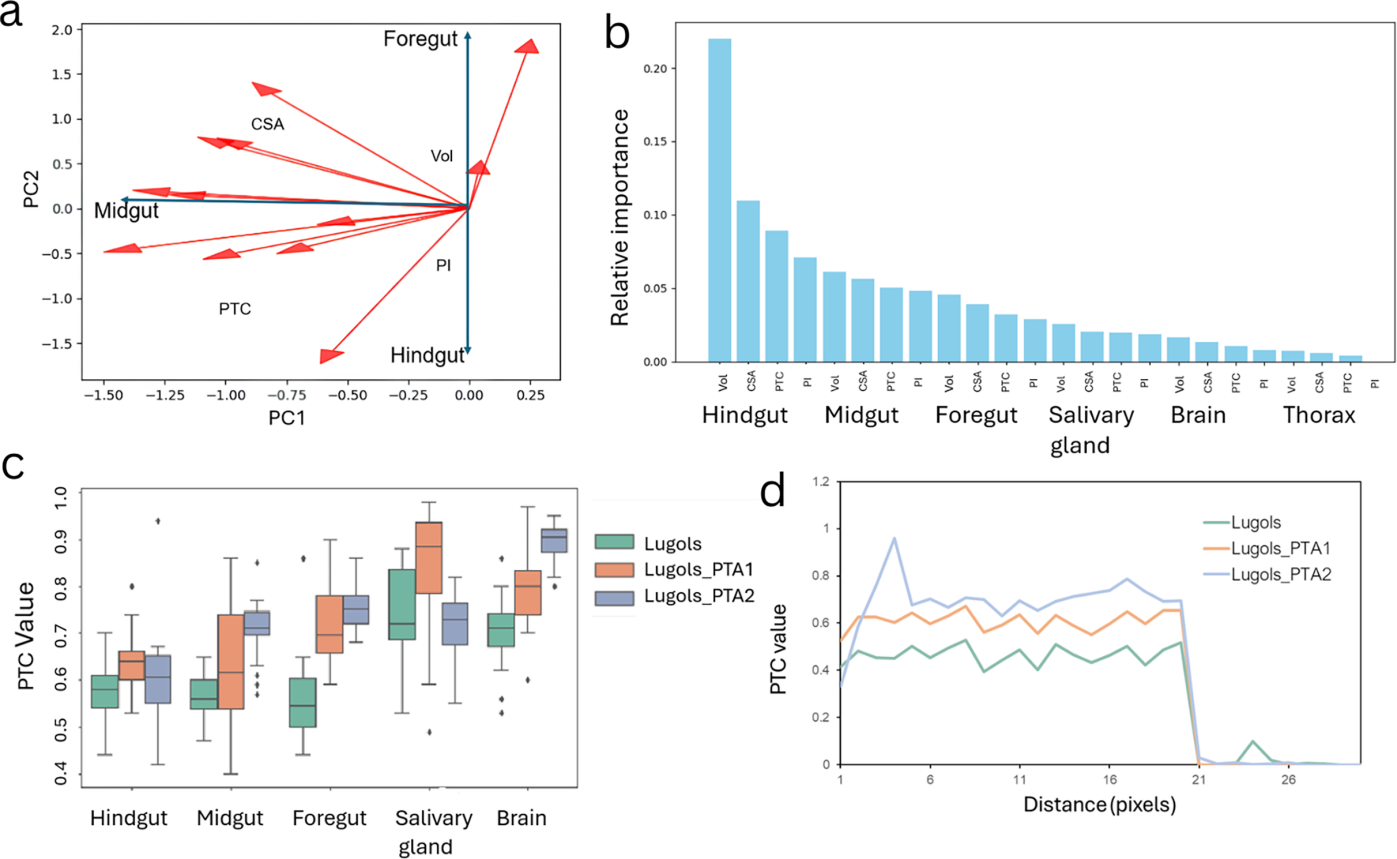
(A) Biplot of Principal Component Analysis (PCA) of termite gut measurements, enabling a visualization of the relationship between the first two principal components (PC1 and PC2) derived from measurements of different gut structures under different staining treatments. Arrows represent the variable contribution to the principal components, with longer arrows indicating stronger influence. The abbreviations pertaining to each measurement are: CSA, cross-sectional area; PI, pixel intensity; PTC, probabilistic tissue contrast; Vol, volume. (B) Relative importance of each variable across the dataset determined by Principal Component Analyses. (C) Box plots comparing the median PTC values for each organ across the staining regimes. The line represents the median value, the whiskers the range (maximum to minimum), the boxes the first quartile to third quartile values, and the dots are outliers. (D) Comparison of PTC plotted against scan distance in pixels for each of the staining regimes. PTC values above 0.6 are considered resolvable.

PC1 and PC2 together account for 37% (with PC1 accounting for 24% and PC2 accounting for 13%) of the variance in the dataset. These loadings suggested there were distinct clustering patterns of organ features based on staining type (Table S1, Fig. 4A). Importance analysis of the various features revealed that the measurements for the hindgut, midgut and foregut were by far of greatest importance (~85–90% importance, see Fig. 4B).

Notably, of the measurements related to hindgut and midgut structures, the cross-sectional area and probabilistic tissue contrast (PTC) measurements of the hindgut and volume and pixel intensity of the midgut showed strong contributions toward PC1, thus indicating that these structures were particularly sensitive to staining differences. The separation along PC2 suggests that foregut and associated glandular structures, such as foregut cross sectional area and PTC, and salivary gland cross sectional area, were also differentially influenced by the staining treatments. The positioning of stain-specific clusters in PCA space suggests that while LS with either 1% or 2% PTA enhanced the resolvability of many gut regions, it may have potentially obscured others, depending largely on tissue composition and radiodensity differences. Such results indicate that different stains may optimize the visualization of specific gut compartments rather than providing uniform enhancement across all measured structures. Further comparative testing was thus done to identify the specific staining treatments that provided for the best differentiation among the distinctive measurements of each of the organs.

### Probabilistic tissue contrasts

Probabilistic Tissue Contrast (PTC) was considered the most important measure of organ resolvability. We therefore examined PTC for each of the organs across treatments and found that for most of organs examined adding 2% PTA to LS enhanced the resolvability of the image by at least as much (or more in the case of the midgut and brain) as when 1% PTA was added. Incidentally, the salivary gland was the only organ where we found that adding 1% PTA to LS enhanced image resolvability more than the addition of 2% PTA (see Fig. 4C, Table S2).

Plotting PTC over pixel distance enabled us to ascertain the influences of staining regime on organ resolvability and whether this was explained by scan duration for the different organs. We found that similarly structured curves were plotted for each of the staining regimes (see Fig. 4D). We thus considered it reasonable to conclude that 2% PTA combines with LS to enhance PTC in the brain and midgut, foregut, and hindgut. Nevertheless, we note that this staining regime was associated with reduced PTC for the salivary gland.

### Pixel intensity

Our analyses found significant differences in image pixel intensity among the organs examined across the three staining regimes (Table S3). We found that using LS plus 2% PTA significantly increased the pixel intensity of the hindgut compared to LS alone and LS plus 1% PTA. A similar trend was observed when we examined the midgut, where LS plus 2% PTA produced images of the highest pixel intensity, followed by LS plus 1% PTA. While specimens stained with LS alone had the lowest pixel intensity. In contrast, similar pixel intensities were found for the foregut when specimens were stained with LS alone and LS plus 1% PTA, while LS plus 2% PTA yielded noticeably greater pixel intensities. Examination of the salivary glands found a similar pattern, with LS plus 2% PTA facilitating the generation of images of greater pixel intensities, while staining regime had no discernible effect on the resultant pixel intensity for brain regions across scans.

### Organ cross sectional areas and volumes

Staining regime influenced the cross-sectional area estimates (Table S4). Using LS plus 2% PTA resulted in greater estimates of cross-sectional areas for the hindgut, midgut, salivary glands, and brain compared to using LS alone and LS plus 1% PTA. However, no significant difference in cross-sectional area estimates was observed for images of the foregut across staining regimes. Such findings aligned with our expectation that LS plus 2% PTA would be the most effective stain for enhancing the resolvability of gastrointestinal features of termites.

We estimated greater volumes for both the hindgut and salivary glands for images of specimens stained with LS plus 2% PTA, while we estimated the greatest midgut and foregut volumes for the images of specimens stained with LS plus 2% PTA and LS plus 1% PTA compared to LS alone. Our volume and pixel intensity estimates remained consistent across all staining regimes for the brain (Table S5).

Taken together our results suggested that, due to the low radiodensity of the digestive organs, the images were at the resolvability limits for µ-CT imaging, and the staining regime played a critical role in enhancing their dimensional resolvability. Most notably, Lugol’s staining plus 2% PTA resolved the majority of the termite’s organs better than the Lugol’s staining alone or when Lugol’s stain was combined with 1% PTA.

## Discussion

Detailed morphometric measurements and analyses of the termite digestive organs are crucial for understanding how such structures support the termite’s microbial biota, and hence its cellulose digestive efficiency and nutrient recycling. Given that insect digestive tissues are notoriously difficult to resolve using conventional micro-computed tomography (μ-CT) due to their extremely low radiodensity, there is an urgent need to find staining methods that provide clear, quantifiable, visualizations of the termite gut. We investigated herein whether the addition of 1% or 2% phosphotungstic acid (PTA) to Lugol’s solution (LS) stain enhanced the contrast and structural differentiation of the hindgut, midgut, foregut, salivary glands, and cephalic ganglion tissues when imaged from µ-CT scans. We found that, under the staining regime of adding 2% PTA to LS, organ resolvability was enhanced. This was evidenced by our measures of pixel intensity and probabilistic tissue contrast (PTC) of the digestive organs across staining regimes. There was also a noticeable and consistent reduction in measurement error when 2% PTA was added to LS. We thus concluded that adding 2% PTA to the Lugol’s stain significantly enhanced image resolution while reducing the background noise encountered when measuring µ-CT imaged termite organs.

Adding 2% PTA to the Lugol’s stain nonetheless did not always result in an adequate reduction of background noise. There was high variability in organ resolvability among the three staining methods, which was probably an artifact of high variability in the composition and radiodensity of the constituent tissues (Bartling et al., 2007; Hanus et al., 2007; Karch et al., 2016; Sansom et al., 2022). Sansom et al. (2022) found, sourcing the same µ-CT images, that the combination of Lugol’s iodine and 1% PTA could significantly enhance the signal-to-noise ratio when examining the haemolymph chamber, cuticle, and SGO in the legs of *N. exitiosis*. This enhancement was attributable to a high affinity for the stains by these tissues resulting in an increase in X-ray attenuation (Koç et al., 2019; Sansom et al., 2022).

Lugol’s iodine binds to glycogen and other iodine-reactive components within soft tissues to provide strong electron density contrasts that facilitate clearer differentiation of μ-CT imaged anatomical structures (Doost & Arnolda, 2021). PTA is a heavy metal stain, so interacts with proteins and lipids, which further enhances the contrast and reduces the background noise by stabilizing and densifying the tissue and enhancing X-ray attenuation (Koç et al., 2019). Combining the two stains accordingly allows for more reliable and accurate imaging and better resolution of fine anatomical details in these exceptionally small animals. Studies using tadpoles (Krings et al., 2017) suggest that the additive effect of combining PTA and Lugol’s stains produces clearer visualization of soft tissues. However, it has been disputed whether just adding 1% PTA to LS is sufficient to improve the image clarity of µ-CT scans of exceptionally small and/or low radiodensity animals (Doost & Arnolda, 2021; Sansom et al., 2022).

In other morphological studies of small insect tissues using µ-CT, a PTC value above 0.6 can be considered sufficient to distinguish fine structural details such as epithelial boundaries, gut lumen delineation, and compartmental tissue densities (Smith et al., 2016, 2020). Our results therefore seem to suggest that Lugol’s solution alone was inadequate for producing a clear differentiation of the hindgut, midgut, and foregut boundaries. When we plotted PTC against pixel distances we found that combining 2% PTA with LS significantly enhanced tissue contrast beyond that achieved with LS alone, or that achieved by LS plus 1% PTA. Our analyses also suggested that at relatively small scan durations, *i.e*., those of 20 pixels or less, only when using LS plus 2% PTA staining can we clearly positively discriminate among the different gastrointestinal structures.

Taken together, our findings suggest that for imaging and measuring termite gastrointestinal tissue from µ-CT scans only Lugol’s stain with the addition of 2% PTA can produce a sufficiently visualizable contrast of gut structures. We nevertheless found the salivary glands and foregut to often remain inadequately resolved even after applying 2% PTA, probably because the tissue composition and/or spatial packing in these regions limits stain penetration (Koç et al., 2019). Accordingly, further optimization may be necessary for complete gastrointestinal system analysis when scanning exceptionally small termites, such as *N. exitiosus*. The PTC of the salivary gland and cephalic ganglion were well above 0.6 whenever Lugol’s stain was combined with at least 1% PTA. Thus, suggesting that these organs have higher radiodensity than the gut so are more readily identified from μ-CT scans using rudimentary staining. Our findings hence showed that staining performance is tissue-specific rather than being universally applicable.

## Conclusions

We demonstrated here that µ-CT imaging can be effectively used to visualize the highly folded gut morphology of a termite with protist-independent digestion, *Nasutitermes exitiosus*. The choice of staining regime however significantly influences the resolvability of their gut and other organs. We found that adding of 2% PTA to LS was necessary to enable a sufficient resolution of the termite’s digestive organs for making reliable morphological measurements. Even then, scan dimensions of around 20 pixels seems to be necessary to achieve a PTC of greater than 0.6, the resolution threshold for distinguishing the required structural details. Other tissue structures, *e.g*., salivary gland and cephalic ganglion, seemed to absorb the stains at different rates, which likely influences the quality of the measurements attained for them using μ-CT imagery.

The novelty of our study was the application of staining with μ-CT imagery to visualise and make accurate and reliable morphological measurements of the digestive tract of a termite as it would naturally appear. Our findings suggest that optimizing the stain-to-tissue ratio is critical for improving reproducibility and clarity in µ-CT studies of exceptionally small insects. Our findings highlight the importance of stain choice in enhancing the visualization of low radiodensity tissues. They also provide a practical framework for future research into the digestive physiology and nutrient processing in termites and other small insects.

## Supplemental Information

10.7717/peerj.21334/supp-1 Supplemental Information 1 Supplemental Figures Tables. All exploratory data analyses and statistical analyses of datasets generated from image measurements.

10.7717/peerj.21334/supp-2 Supplemental Information 2 Raw data.

## Additional Information and Declarations

### Data Availability

The following information was supplied regarding data availability:

The data is available at Zenodo: Blamires, S. (2026). Micro-CT-Scans-of-termite-guts [Data set]. In PeerJ. Zenodo. https://doi.org/10.5281/zenodo.18995736.
