# Supplementary material for: Micro-computed tomography of termite gut anatomy: effects of staining regime on resolvability
Source: PeerJ. 2026 Jul 3;14:e21334. doi: 10.7717/peerj.21334 (PMC13335497; doi:10.7717/peerj.21334)
Supplement: Supplemental Information 1 — All exploratory data analyses and statistical analyses of datasets generated from image measurements. [file peerj-14-21334-s001.zip › Blamires et al_Supplemental Material.pdf]

Supplemental Materials

Micro-Computed Tomography of Termite Gut Anatomy: Effects of Staining Regime on Organ Resolvability

Sean J Blamires[1,2], Travers Sansom[1], Sebastian Oberst[1]

[1]Centre for Audio, Acoustics and Vibration, School of Mechanical and Mechatronic Engineering, University of Technology, Sydney, NSW 2007, Australia

[2]School of Biological, Earth and Environmental Science, University of New South Wales, Sydney, NSW 2052, Australia

Supplemental Figures

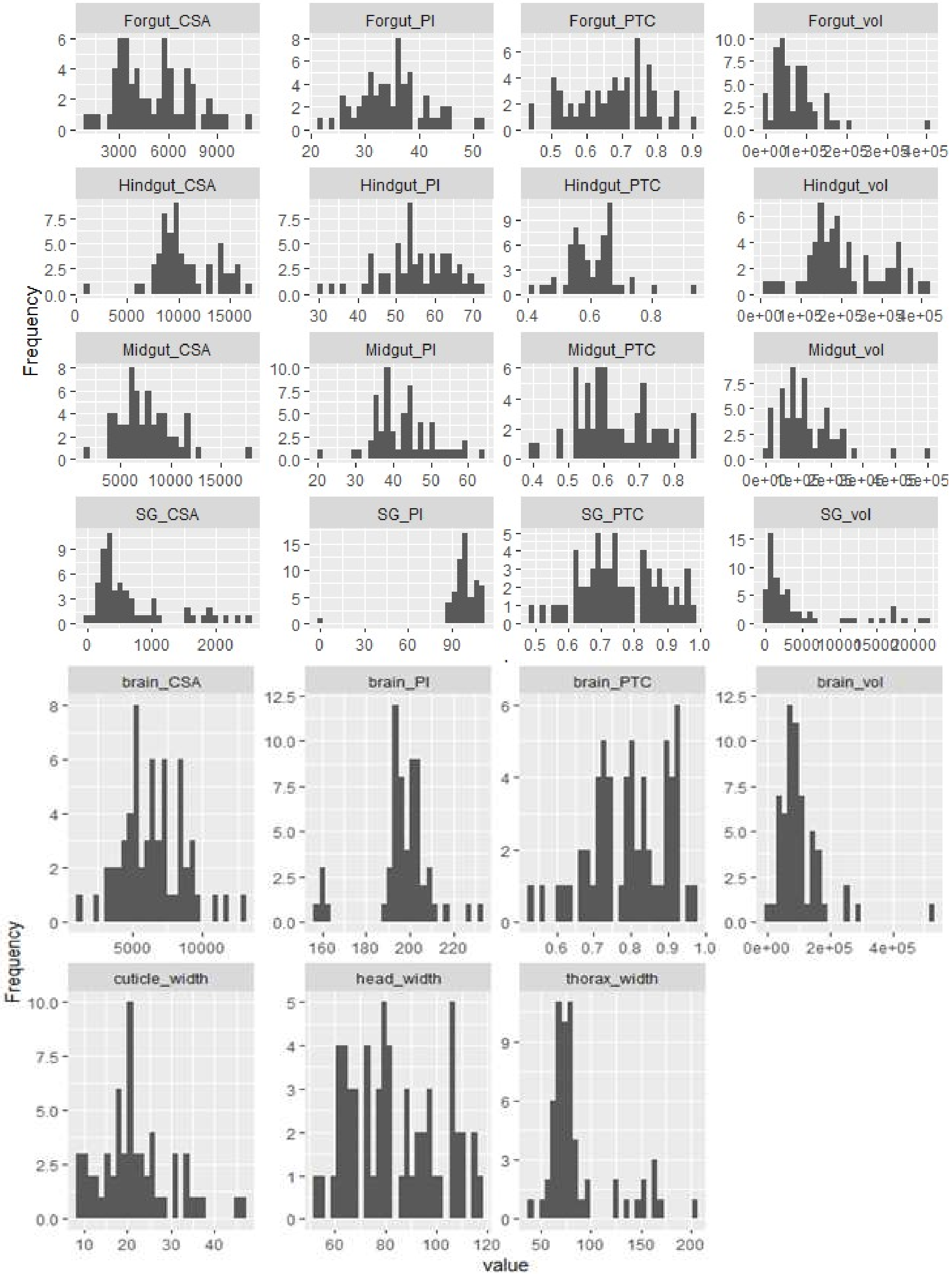

Figure S1. Histograms of the data distribution of all of the variables within the dataset.

The individual measurements that are plotted are: forgut_CSA = cross-sectional area of the forgut, forgut_PI = pixel intensity of the forgut,, forgut_PTC = probabilistic tissue contrast of the forgut, forgut_vol = forgut volume, midgut_CSA = cross-sectional area of the midgut, midgut_PI = pixel intensity of the midgut, midgut_PTC = probabilistic tissue contrast of the midgut, midgut_vol = midgut volume, hindgut_CSA = cross-sectional area of the hindgut, hindgut_PI = pixel intensity of the hindgut, hindgut_PTC = probabilistic tissue contrast of the hindgut, hindgut_vol = hindgut volume, SG_CSA = cross-sectional area of the salivary glands, SG_PI = pixel intensity of the salivary glands, SG_PTC = probabilistic tissue contrast of the salivary glands, SG_vol = salivary glands volume, brain_CSA = cross-sectional area of the brain, brain_PI = pixel intensity of the brain, brain_PTC = probabilistic tissue contrast of the brain, SG_vol = brain volume, cuticle_width = width of the cuticle layer, head width = maximum width of the head, and thorax width = maximum width of the thorax.

From this plot, it was ascertained that the distributions of all variables were non-normal meaning frequency-independent statistics were required to compare the effects of the different staining regimes between any of the parameters.

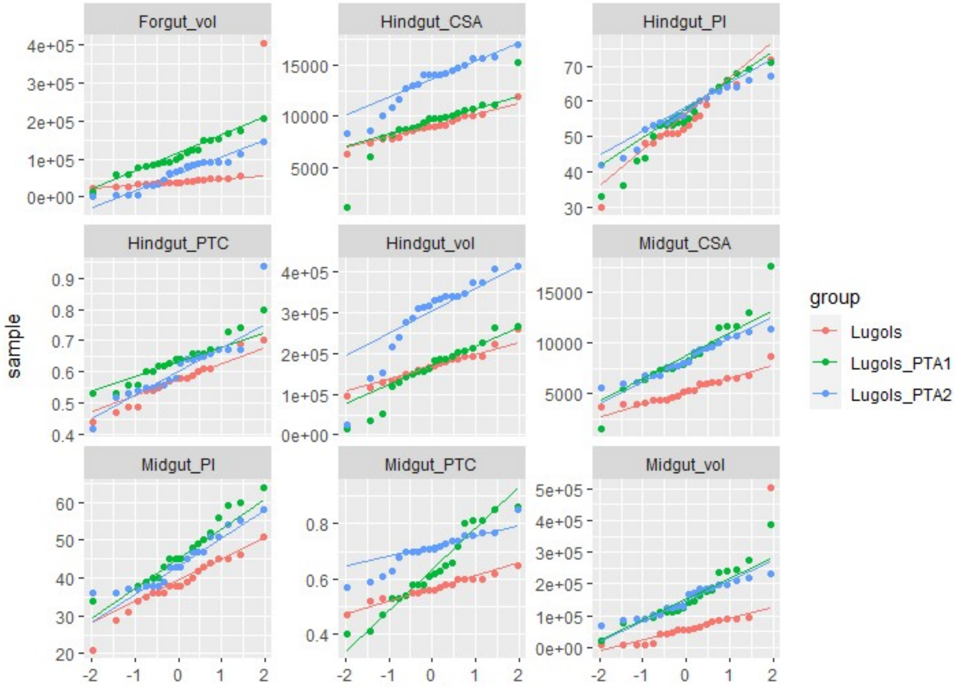
Forgut_vol
Hindgut_CSA
Hindgut_PI
Hindgut_PTC
Hindgut_vol
Midgut_CSA
Midgut_PI
Midgut_PTC
Midgut_vol
sample
group
Lugols
Lugols_PTA1
Lugols_PTA2

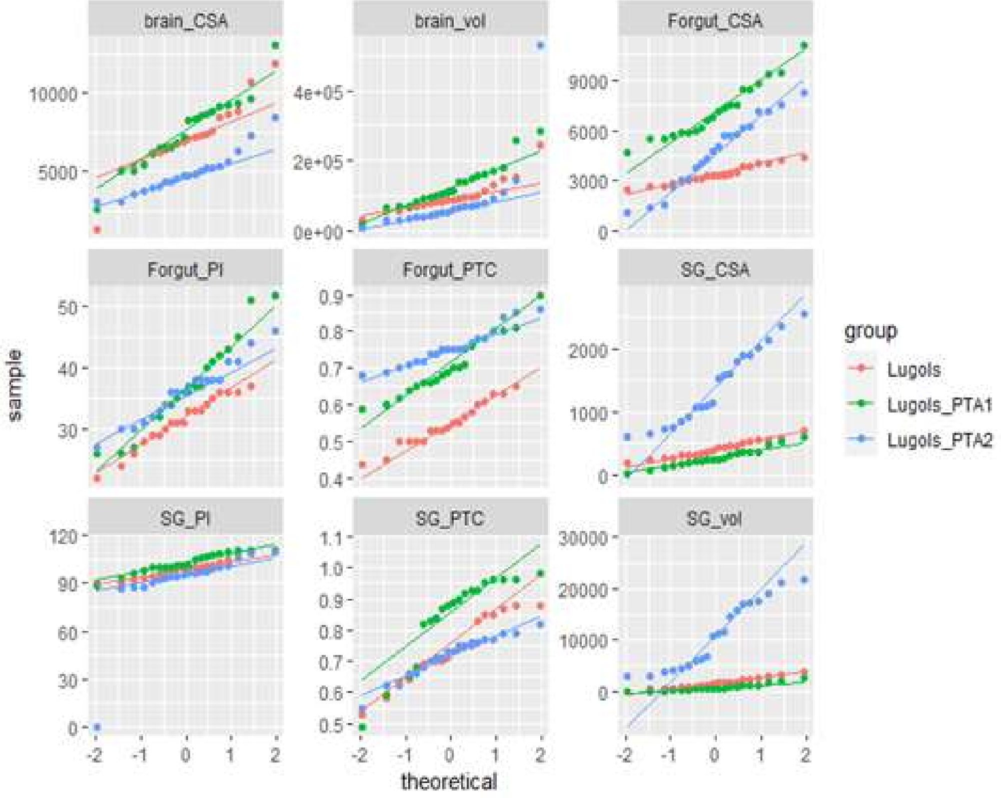
brain_CSA
brain_vol
Forgut_CSA
Forgut_PI
Forgut_PTC
SG_CSA
SG_PI
SG_PTC
SG_vol
sample
theoretical
group
Lugols
Lugols_PTA1
Lugols_PTA2

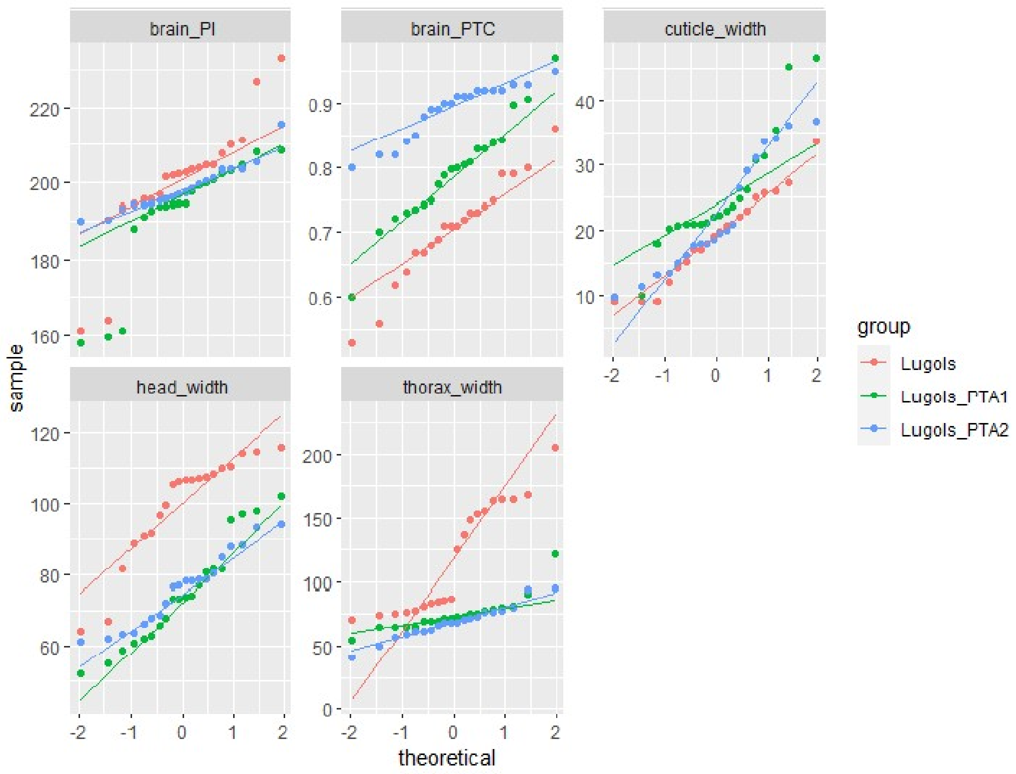

Figure S2. Quantile-quantile plots to assess whether the data can be attributed to the same distribution across staining regimes.

The individual measurements that are plotted are: forgut_CSA = cross-sectional area of the forgut, forgut_PI = pixel intensity of the forgut,, forgut_PTC = probabilistic tissue contrast of the forgut, forgut_vol = forgut volume, midgut_CSA = cross-sectional area of the midgut, midgut_PI = pixel intensity of the midgut, midgut_PTC = probabilistic tissue contrast of the midgut, midgut_vol = midgut volume, hindgut_CSA = cross-sectional area of the hindgut, hindgut_PI = pixel intensity of the hindgut, hindgut_PTC = probabilistic tissue contrast of the hindgut, hindgut_vol = hindgut volume, SG_CSA = cross-sectional area of the salivary glands, SG_PI = pixel intensity of the salivary glands, SG_PTC = probabilistic tissue contrast of the salivary glands, SG_vol = salivary glands volume, brain_CSA = cross-sectional area of the brain, brain_PI = pixel intensity of the brain, brain_PTC = probabilistic tissue contrast of the brain, SG_vol = brain volume, cuticle_width = width of the cuticle layer, head width = maximum width of the head, and thorax width = maximum width of the thorax.

From this plot it was ascertained that there was high distributional variability in the data which needed to be accounted for in the subsequent between treatment (staining) analyses.

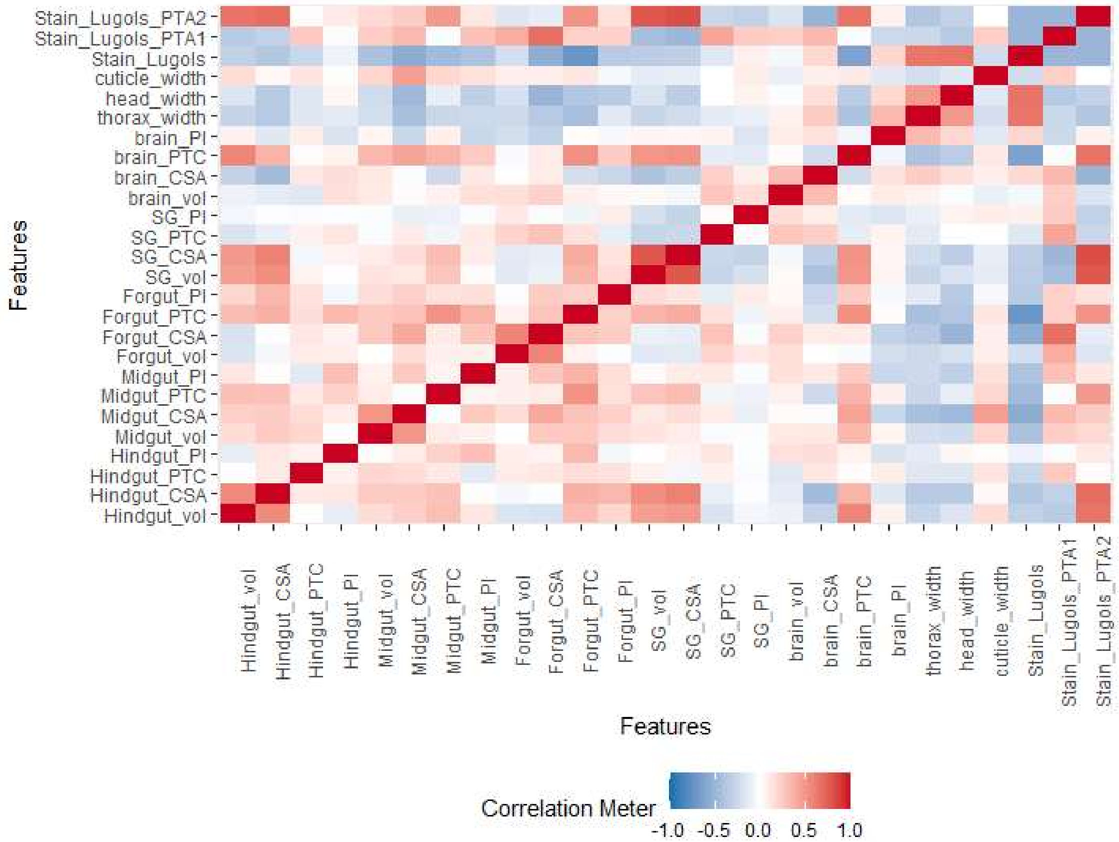

Figure S3. Pearson's correlations between the variables in the dataset generated. The z-axis colour bar was not included, and the correlation values were represented as running from red (strong positive correlation) to blue (strong negative correlation).

The individual measurements that are plotted are: forgut_CSA = cross-sectional area of the forgut, forgut_PI = pixel intensity of the forgut,, forgut_PTC = probabilistic tissue contrast of the forgut, forgut_vol = forgut volume, midgut_CSA = cross-sectional area of the midgut, midgut_PI = pixel intensity of the midgut, midgut_PTC = probabilistic tissue contrast of the midgut, midgut_vol = midgut volume, hindgut_CSA = cross-sectional area of the hindgut, hindgut_PI = pixel intensity of the hindgut, hindgut_PTC = probabilistic tissue contrast of the hindgut, hindgut_vol = hindgut volume, SG_CSA = cross-sectional area of the salivary glands, SG_PI = pixel intensity of the salivary glands, SG_PTC = probabilistic tissue contrast of the salivary glands, SG_vol = salivary glands volume, brain_CSA = cross-sectional area of the brain, brain_PI = pixel intensity of the brain, brain_PTC = probabilistic tissue contrast of the brain, SG_vol = brain volume, cuticle_width = width of the cuticle layer, head width = maximum width of the head, and thorax width = maximum width of the thorax.

This analysis showed that the data for cross-sectional area and volume, PTC and pixel intensity were autocorrelated for each of the anatomical features analysed.

Supplemental Tables

Table S1. Principal component (PC) scores for the first two principal components (PC1 and PC2), which capture the most significant variance in gut structure measurements across different staining treatments. PC1 primarily reflects variations in overall tissue volume and cross-sectional area, while PC2 captures differences related to tissue contrast and proportional thickness. Higher PC1 scores indicate samples with larger gut structures, while variation along PC2 suggests differences in how staining affects tissue discernibility.

PC1	PC2
3.320245	6.259882
-0.286118	0.001672
2.782610	-0.105977
2.445011	-0.079856
1.652252	-1.379476

Table S2. Results of a Kruskall-Wallis multi-variate tests of median PTC values across staining regimes, and the results of the subsequent Dunn's test wherever statistical significance was indicated. Staining regimes: PTA2 = Lugol's solution with 2% PTA, PTA 1 = Lugol's solution with 2% PTA, Lugols = Lugol's solution alone.

	Organ				
Kruskall-Wallis statistics	forgut	hindgut	midgut	Salivary gland	brain
χ^2	8.475	5.091	15.838	25.88	15.021
df	2	2	2	2	2
P	0.022	0.078	0.011	<0.001	0.014
Dunn's test	PTA2=PTA1>Lugols	--	PTA2>PTA1=Lugols	PTA1>PTA2>Lugols	PTA2>PTA1>Lugols

Table S3. Results of multivariate analyses of variance (MANOVA) comparing the pixel intensity measured for images generated across the three staining regimes.
Table (a) shows the results for four multivariate test statistics (Wilk's λ, Pillai's Trace, Hotelling-Lawley Trace, and Roy's Greatest Root) with each found differences in image resolvability across organs (P-values <0.05).
Table (b) shows the results of Tukey's post-hoc comparison of the mean values for each organ examined across the three staining regimes, where: L = staining was with Lugol's Iodine solution (LS) alone, L-PTA1 = staining with LS plus 1% phosphotungstic acid (PTA), and L-PTA2 = staining with LS plus 2% PTA.

a)

	Value	df	F-value	P-value
Intercept		5,53	1692.030	<0.001
Wilk's λ	0.631	10,106	2.742	0.004
Pillai's Trace	0.403	10,108	2.733	0.004
Hotelling-Lawley Trace	0.528	10,76.8	2.770	0.006
Roy's Greatest Root	0.384	5,54	4.155	0.003

b)

Organ	Tukey's HSD result
forgut	L < L-PTA1 = L-PTA2
hindgut	L = L-PTA1 = L-PTA2
midgut	L < L-PTA1 < L-PTA2
salivary gland	L = L-PTA1 < L-PTA2
brain	L = L-PTA1 = L-PTA2

Table S4. Results of multivariate analyses of variance (MANOVA) comparing the measured organ cross sectional area for images generated across the three staining regimes.
Table (a) shows the results for four multivariate test statistics (Wilk's λ, Pillai's Trace, Hotelling-Lawley Trace, and Roy's Greatest Root) with each showing differences in image resolvability across organs (P-values <0.05).
Table (b) shows the results of Tukey's post-hoc comparison of the mean values for each organ examined across the three staining regimes, where: L = staining was with Lugol's Iodine solution (LS) alone, L-PTA1 = staining with LS plus 1% PTA, and L-PTA2 = staining with LS plus 2% PTA.

a)

	Value	df	F-value	P-value
Intercept		5,53	183.572	<0.001
Wilk's λ	0.151	10,106	16.613	<0.001
Pillai's Trace	1.029	10,108	13.011	<0.001
Hotelling-Lawley Trace	3.979	10,76.8	20.835	<0.001
Roy's Greatest Root	3.251	5,54	38.281	<0.001

b)

Organ	Tukey's HSD result
forgut	L = L-PTA1 = L-PTA2
hindgut	L = L-PTA1 < L-PTA2
midgut	L < L-PTA1 = L-PTA2
salivary gland	L = L-PTA1 < L-PTA2
brain	L = L-PTA1 < L-PTA2

Table S5. Results of multivariate analyses of variance (MANOVA) comparing the organ volumes measured for images generated across the three staining regimes.
Table (a) shows the results for four multivariate test statistics (Wilk's λ, Pillai's Trace, Hotelling-Lawley Trace, and Roy's Greatest Root) with each found differences in image resolvability across organs (P-values <0.05).
Table (b) shows the results of Tukey's post-hoc comparison of the mean values for each organ examined across the three staining regimes, where: L = staining was with Lugol's Iodine solution (LS) alone, L-PTA1 = staining with LS plus 1% PTA, and L-PTA2 = staining with LS plus 2% PTA.

a)

	Value	df	F-value	P-value
Intercept		5,53	25.556	<0.001
Wilk's λ	0.160	10,106	15.887	<0.001
Pillai's Trace	1.127	10,108	13.942	<0.001
Hotelling-Lawley Trace	3.451	10,76.8	18.071	<0.001
Roy's Greatest Root	2.814	5,54	30.931	<0.001

b)

Organ	Tukey's HSD result
forgut	L < L-PTA1 = L-PTA2
hindgut	L = L-PTA1 < L-PTA2
midgut	L < L-PTA1 = L-PTA2
salivary gland	L = L-PTA1 < L-PTA2
brain	L = L-PTA1 = L-PTA2
